# Spectroscopic Properties of Si-nc in SiO_x_ Films Using HFCVD

**DOI:** 10.3390/nano10071415

**Published:** 2020-07-20

**Authors:** Zaira Jocelyn Hernández Simón, Jose Alberto Luna López, Alvaro David Hernández de la Luz, Sergio Alfonso Pérez García, Alfredo Benítez Lara, Godofredo García Salgado, Jesus Carrillo López, Gabriel Omar Mendoza Conde, Hayde Patricia Martínez Hernández

**Affiliations:** 1 Centro de Investigaciones en Dispositivos Semiconductores (CIDS-ICUAP), Benemérita Universidad Autónoma de Puebla (BUAP), Col. San Manuel, Cd. Universitaria, Av. San Claudio y 14 Sur, Edificios IC5 y IC6, Puebla 72570, Mexico; imezaira@gmail.com (Z.J.H.S.); joalvada1@hotmail.com (A.D.H.d.l.L.); godgarcia@yahoo.com (G.G.S.); jesus.jecarril@gmail.com (J.C.L.); gaomec13@gmail.com (G.O.M.C.); 2 Centro de Investigación en Materiales Avanzados (CIMAV), Alianza Norte 202, Parque de Investigación e innovación Tecnológica Apodaca, Nuevo León 66600, Mexico; alfonso.perez@cimav.edu.mx; 3 CONACYT-Centro de Investigaciones En Óptica AC (CIO), León, Guanajuato 36000, Mexico; alfredbl@cio.mx; 4 Departamento de Ingeniería Eléctrica y Electrónica, Instituto Tecnológico de Apizaco (ITA), Fco I Madero s/n, Barrio de San José, Apizaco, Tlaxcala 90300, Mexico; pathaymh@yahoo.com

**Keywords:** SiO_x_ films, Si-ncs, band gap engineering, spectroscopic characterizations, ellipsometric spectroscopy, photoluminescence

## Abstract

In the present work, non-stoichiometric silicon oxide films (SiO_x_) deposited using a hot filament chemical vapor deposition technique at short time and simple parameters of depositions are reported. This is motivated by the numerous potential applications of SiO_x_ films in areas such as optoelectronics. SiO_x_ films were characterized with different spectroscopic techniques. The deposited films have interesting characteristics such as the presence of silicon nanoclusters without applying thermal annealing, in addition to a strong photoluminescence after applying thermal annealing in the vicinity of 1.5 eV, which may be attributed to the presence of small, oxidized silicon grains (less than 2 nm) or silicon nanocrystals (Si-nc). An interesting correlation was found between oxygen content, the presence of hydrogen, and the formation of defects in the material, with parameters such as the band gap and the Urbach energies. This correlation is interesting in the development of band gap engineering for this material for applications in photonic devices.

## 1. Introduction

The emission of visible light in nanostructured oxides (applicable to optoelectronic devices) [1,2,3], light absorption effects such as the so-called down conversion effect (solar cells) [4,5], and resistive switching (non-volatile memories of multiple states) [6,7,8,9] are some of the characteristics that make SiO_x_ a material that needs to be studied deeply, also taking into account that silicon is the second most abundant material on our planet. Another favorable feature that this material provides is that it can be obtained using multiple synthesis techniques [10,11,12,13,14]. Among these techniques, it has been found that the technique of chemical vapor deposition activated using a hot filament (HFCVD) offers important characteristics due to the ease of obtaining thin films and powders with diverse electrical and optical characteristics. The HFCVD technique consists of a chemical process that uses high purity molecular hydrogen as a reagent, which is dissociated to work in its atomic form. Such dissociation is achieved thermally through an incandescent filament at ~2000 °C; the chemical reaction of this process is described in references [15,16].

Although there are several reports of the behavior of this material using various techniques [10,11,12,13,14,15], in this investigation the spectroscopic and compositional behavior of SiO_x_ films are analyzed, where the material is obtained using the HFCVD technique with a short deposition time of 3 min. This material is suitable for large-scale applications and may be important for future applications, such as light absorbing devices (photosensors and solar cells), as well as for light emitting devices.

In addition, in this research we try to explain phenomena such as the characteristic light emission of SiO_x_ in approximately 1.5 eV that appears when the material has been annealed, which has been attributed to phenomena such as the quantum confinement that is presented by a phase separation and formation of nanocrystals or due to the formation of various defects in the material; however, no agreement has been reached between these theories. An analysis of how deposit conditions affect optical properties of great technological interest such as the band gap is also carried out.

## 2. Materials and Methods

The SiO_x_ films were deposited on p type silicon substrates with a diameter of 2 in, orientation (1 0 0), and resistivity of 1–5 ohm-cm. Four SiO_x_ films were deposited using the HFCVD technique, the parameters that were changed during the deposit process were the distance from source to substrate (ssd) (5 mm and 8 mm) and the hydrogen flux levels (100 sccm and 25 sccm), whereas the parameters that were kept constant are the deposit time (3 min), the voltage applied to the filaments (74 V), the filament-to-source distance (fsd) (6 mm), and the system pressure (1 atm). A thermal annealing was subsequently applied to the films obtained, which was applied at 1050 °C. This temperature is used because it has been reported that after thermal annealing at temperatures above 1000 °C, SiO_x_ reacts to produce silicon nanocrystals or nanoclusters and structures with different oxidation states with or without defects [17,18,19]. This thermal annealing was carried out in a tubular furnace with a nitrogen flowing environment for 1 hour. In total, eight films were obtained; their labeling is described in Table 1.

Regarding the chosen parameters for the deposit, it is important to consider that both the deposit temperature and the hydrogen gas flow level are important in the formation of thin films. The deposit temperature is regulated by the distance between the source and substrate, while the filament-to-source distance regulates the number of precursors.

**Figure 1:**
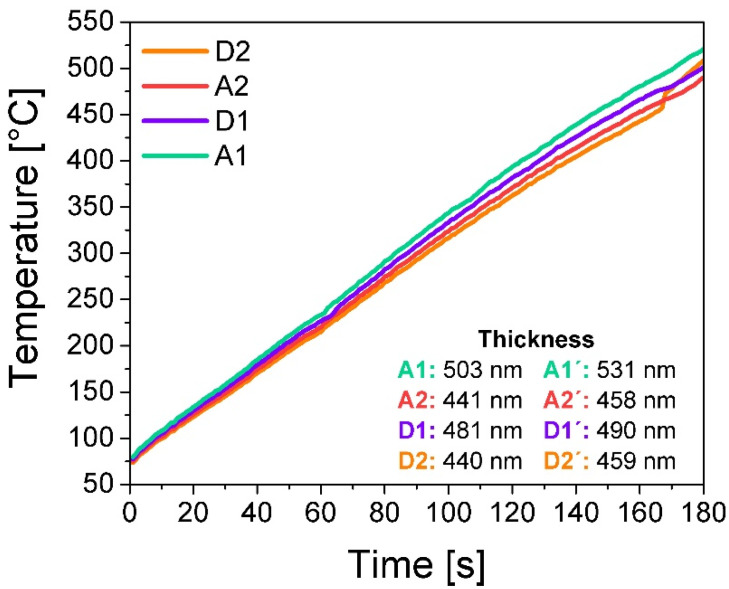
Substrate temperature during SiO_x_ film deposition and thickness reached for each film.

The temperatures reached for the substrate during the deposit are shown in Figure 1, as well as the thicknesses obtained, which were measured using profilometry. The trend in the temperatures reached by the substrate follows what was mentioned regarding the distance from source to substrate, while at a lower flow the substrate temperature increases. The temperature follows a linear trend for all films; however, in the case of film, D2 is disturbed in the last seconds of deposit. This could be caused by the buckling of some filament causing a decrease in the filament-to-source distance, increasing temperature. In the case of the thicknesses, these increase with annealing. This abnormal behavior could be explained as an additional formation of oxide caused by diffusion of residual O_2_ as well as interstitial, as mentioned in [20], where despite performing a heat treatment in an Ar environment, there is an increase in thickness.

To obtain the optical characteristics of the deposited SiO_x_ films, measurements of the refractive index (n) and extinction coefficient (k) were performed using spectroscopic ellipsometry on a Horiba UVISEL ellipsometer with a spectral range of (0.6–4.8 eV) and incidence angle of 70°. In addition, the IR absorption spectra (FTIR) of the SRO films were obtained with a Bruker Vector 22 spectrometer in a range from 4000 cm^−1^ to 400 cm^−1^ with a resolution of 1 cm^−1^. The photoluminescence (PL) spectra were also measured with a FluroMax 3 Horiba Jobin Yvon Spectrofluorometer with a 150 W xenon excitation lamp, 0.3 nm resolution, range from 370 to 1000 nm, and high sensitivity emission detector. The excitation line used to obtain the photoluminescence spectra was 335 nm. In addition, XPS measurements were made using an Escalab250Xi Thermo Scientific spectrometer using a monochromatic Al XR15 X-ray source. The depth profile was acquired using Ar^+^ etching. The beam energy was 4 keV, and the sputter current was 2.5 μA. The size of the raster was approximately 15 times larger than the XPS-measured area. Finally, micrographs were obtained using High Resolution Transmision Electronic Microscopy (HRTEM) in Nanotech JEOL JEM-2200FS + Cs equipment with a spherical aberration corrector on the condenser lens and operated at an acceleration voltage of 200 kV.

## 3. Results

The spectroscopic ellipsometry technique allows the optical parameters of interest to be known by obtaining the change in the amplitude of the light after reflection (Ψ) and the phase change of this (Δ), through the mathematical inversion of the data (Ψ, Δ), which are converted directly into the optical constants refractive index (n) and extinction coefficient (k) [21]. With the values n and k obtained by spectroscopic ellipsometry the transmittance and reflectance of all deposited SiO_x_ films were calculated by means of the reflection and transmission coefficients for the s and p polarizations which were calculated using Snell’s law [22].

**Figure 2:**
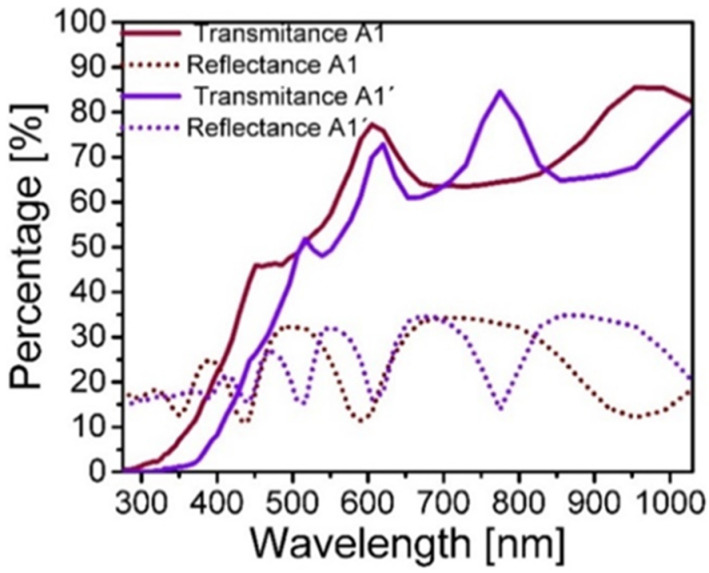
Reflectance and transmittance of the A1, A1’ SiO_x_ films without (A1) and with thermal annealing (A1’).

**Figure 3:**
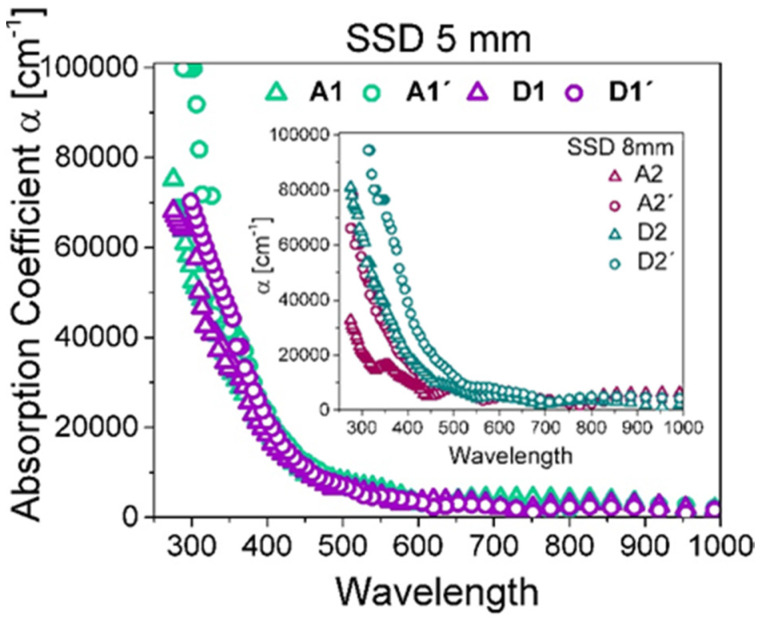
The absorption coefficients of the SiO_x_ films without (A1, A2, D1, D2) and with thermal annealing (A1’, A2’, D1’, D2’) considering two source-to-substrate distances (ssd): 5mm (A1, A1’, D1, D1’) and 8mm (A2, A2’, D2, D2’).

Figure 2 shows the reflectance and transmittance obtained specifically from A1 and A1’ SiO_x_ films. The transmittance and reflectance of the films A1 and A1’ are shown as a sample of the results obtained, however, Figure 3 shows the complete results of the absorption coefficients obtained. In Figure 2, transmittance less than 85% is observed and likewise reflectance less than 35%, in addition to the typical oscillations caused by the interference of the electromagnetic modes confined inside the films [23]. On the other hand, to analyze deeply these results, the absorption coefficient α is calculated using the equations [23]

(1) $$T≅\left(1-R\right)e^{-\alpha t}$$

(2) ln(T)≅ln(1−R)−αt

(3) $$\alpha =\frac{1}{t}ln\frac{\left(1-R\right)}{T},$$

which involve the values of reflectance and transmittance experimentally obtained. Since the specified transmittance and reflectance data have multiple interferences, obtaining the optical absorption coefficient is a process that may include a certain degree of error. For this reason, it is clarified that the values obtained are parameters that describe a particular sample and not the physical property.

The α results calculated for all deposited films are shown in Figure 3, in which the inset shows the absorption coefficients of the films deposited at a higher sdd. From this figure it is observed that the absorption coefficient displays values which are similar to those reported in SiO_x_ films obtained using other methods of analysis, such as the transmittance obtained using UV-visible spectroscopy [16]. It is worth noting that thermal annealed films increase their absorption coefficient and such a tendency prevails in all films, in addition to a slight increase in α for films deposited with a hydrogen flow level of 100 sccm. Such behavior results due to thermal effects provoke an atomic structural rearrangement which enriches the absorption mechanisms inside the material. Although this phenomenon has been previously reported in this material, it is still not well understood what type of arrangement occurs to increase the absorption coefficient when applying thermal annealing. It has been suggested that it may be due to the formation of ultra-small amorphous silicon grains that induce disorder in the network or due to the formation of nanocrystals since the optical properties of bulk and nanoscale materials differ [24], or it has also been suggested that it may be due to an increase in the Si dangling bonds given the hydrogen desorption [23] that is observed later. In the case of the present investigation, the three previously mentioned phenomena occur, for which all of them could contribute to the increase of the absorption coefficient. Theoretical studies have been carried out in other research works [25] where the reactions that occur when applying thermal treatment are modeled.

Using the values of the absorption coefficient (α), we estimate the band gap energy (BG) using the Tauc relation [26] where the BG was obtained using linear regression considering the equation

(4) $${\left(\alpha h\nu \right)}^{1/2}=A\left(h\nu -E_{g}\right)$$

where α is the absorption coefficient, hν is the energy of the impinging photon, $E_{g}$ is the BG, and A is an arbitrary constant. We also choose the exponent ½, which corresponds to an indirect allowed transition according to what other authors have used [27,28] for this material.

As is widely known, an amorphous material exhibits a complex structure of the density of states, such is the case for the SiO_x_ films. For this reason, the analysis of absorption mechanisms in this type of material is so complex; however, we can realize an approximation of the absorption coefficient through the presence of electronic states inside the band gap energy in the proximity of the valence and conduction band edges, as is proposed by the Urbach energies, which is now applied for the deposited SiO_x_ films using Urbach’s empirical rule, given by the equation [29]

(5) $$\alpha =a_{0}exp\frac{\left(hv\right)}{Eu}\;.$$

where α is the absorption coefficient, $a_{0}$ is a constant, and Eu is Urbach’s energy, from (5) we obtain the following equation:

(6) $$ln\;\alpha =ln\;a_{0}+\frac{\left(hv\right)}{UE}$$

The Urbach energy (UE) can be obtained from the inverse slope of the straight line of the path ln α versus hv energy of the incident photon.

**Figure 4:**
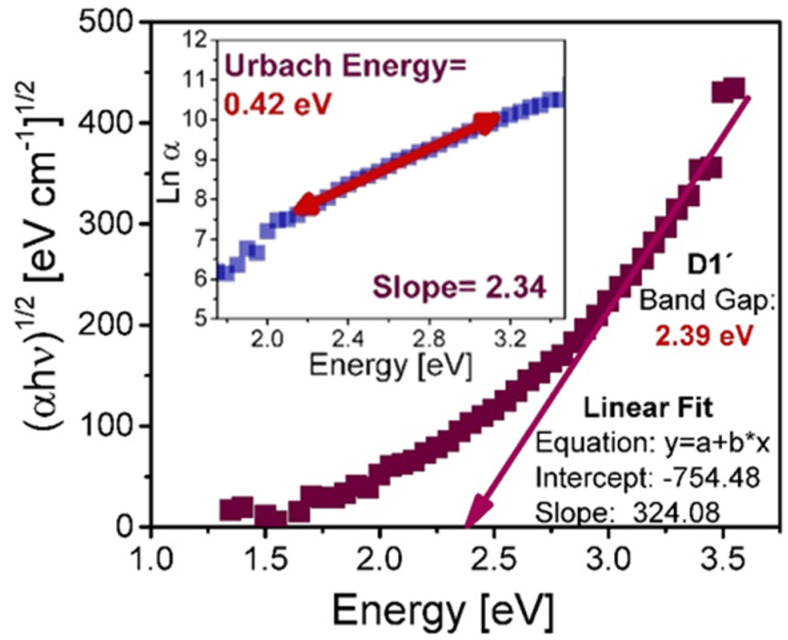
Energy band gap calculated for D1’ film with its corresponding Urbach energy.

Figure 4 shows an example of the procedure used to calculate the BG, while the inset in this figure shows the value of the corresponding UE for D1’ film. To obtain the BG, the linear region belonging to the high absorption edge was taken into account in such a way that in Figure 4 it corresponds to energies from 3 eV to 3.5 eV, while to calculate the UE we utilize the energy region that is below the high absorption edge which corresponds to the energy range from 2.2 eV to 3 eV. This procedure is applied to each of the deposited films, and the results are displayed in Table 2. From the results exhibited in Table 2, it is remarkable that when we have a higher ssd in the deposit or a greater flow of hydrogen the value of BG energy increases and the UE decreases, a trend that is accomplished for all the films except for the D2 film, this could be due to the increase in the temperature of the substrate in the last seconds of deposit of the film D2, whereby there was a change in the deposit conditions, so the trend is not followed.

**Figure 5:**
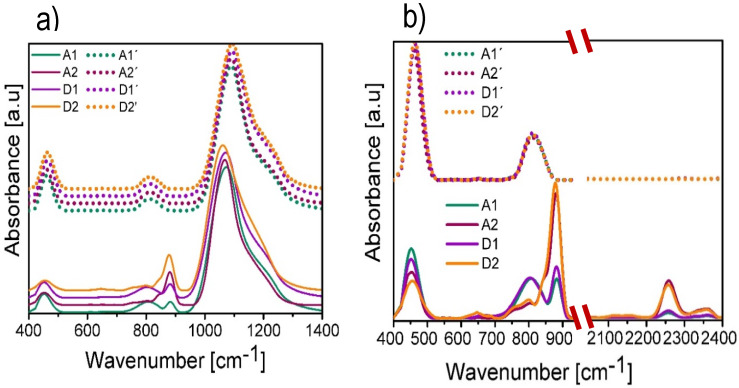
(**a**) The Fourier Transform Infrared (FTIR) spectra obtained for the SiO_x_ films in a range from 400 cm^−1^ to 1400 cm^−1^. (**b**) FTIR spectra obtained for SiO_x_ films in a range from 400 cm^−1^ to 950 cm^−1^ and from 2150 cm^−1^ to 2400 cm^−1^.

As can be seen, in general the thermal annealing increases the BG value and decreases the UE, which is an indication that a structural rearrangement occurs in the atomic lattice. Due to thermal effects such a phenomenon favors the crystallinity of the material. For that reason the UE is reduced, indicating that the amorphous phase is also reduced. This is corroborated using the FTIR technique. Figure 5a shows the FTIR spectra offset in the absorbance axis for all SiO_x_ films in a range from 400 cm^−1^ to 1400 cm^−1^. The solid lines correspond to the FTIR spectra of the films without thermal annealing and the dotted lines to the FTIR spectra with thermal annealing. By virtue of the intensity of the localized peak in the range from 950 cm^−1^ to 1350 cm^−1^, it is difficult to appreciate clearly both the shape and position of the peaks having less intensity. For this reason, in Figure 5b) an amplification of the intensity of these peaks is shown by placing the spectra on a scale from 400 cm^−^¹ to 950 cm^−^¹ and from 2150 cm^−^¹ to 2400 cm^−^¹. All the spectra shown have been normalized with the purpose of not considering the variation of the thickness of the films and only taking into account the variations or shifts in wave number in the spectra.

In the obtained FTIR spectra, the characteristic absorption peaks of SiO_2_ were identified. These peaks correspond to the vibration modes Si–O–Si rocking (R) at 458 cm^−1^, Si–O–Si bending (B) at 812 cm^−1,^ and Si–O–Si stretching (S) at 1082 cm^−1^ [15]. There are shifts in the mentioned peaks indicating a stoichiometry different from that of SiO_2_, which confirms the fact that the films are made up of SiO_x_. The absorption peaks attributed to the Si–H bond were also found in the H–Si≡O_3_ configuration, which are B at 880 cm^−1^ and S at 2250 cm^−1^; in addition, with a lower intensity absorption peaks attributed to the Si-H bond in the H–Si≡Si_3_, H–Si≡O_2_, H–Si≡O_1_ configuration were found, which are S at 2100 cm^−1^, 2156 cm^−1^, and 2119 cm^−1^, respectively. These configurations are reported in the literature [29].

Table 3 shows the positions of the absorption peaks present in the deposited SiO_x_ films as well as the vibrational modes and the type of molecule to which they are attributed.

We can observe that, compared to the peak corresponding to the vibrational mode R of SiO_2_, the peak of the SiO_x_ films without thermal annealing shows a slight shift towards lower wave numbers, this indicates a slight increase in silicon composition in some Si–O bonds, whereas with thermal annealing we have a condition closer to that of the SiO_2_ stoichiometry.

On the other hand, the peak located at 812 cm^−1^ (Si–O–Si B) appears in all films; however, in films without thermal annealing, this peak is attached to the peak located at 880 cm^−1^, which corresponds to the H–Si≡O_3_ B molecule. This peak is closely related to the peak located at 2256 cm^−1^ that corresponds to the vibrational mode S of the H–Si≡O_3_ molecule. Having both vibrational modes in mind we corroborate the existence of the H–Si≡O_3_ configuration in the films without thermal annealing, which is attributed to the hydrogen incorporation in the deposition process. In addition, a stronger intensity of these vibrational modes is observed for the films deposited at a larger ssd. It is also observed that when increasing the H–Si≡O_3_ B peak the Si-O-Si R peak decreases, which is highly linked to the increase of silicon content in the material [30]. After the film is annealed, both peaks disappear due to the desorption of hydrogen at high temperatures and the intensity of the Si–O–Si B mode increases, which is, as already mentioned, characteristic of SiO_2_.

The peak corresponding to the vibrational mode S provides information about the composition of the deposited films. When this peak displaces towards higher wave number values it indicates that the density of the Si–O–Si bonds is increased, and such films are more stoichiometric [31]. In films without thermal annealing, the peak assigned to the vibrational mode of stretching is shifted towards smaller wave numbers, suggesting a higher proportion of silicon in the amorphous phase and a greater amount of unbound oxidation states. However, when applying thermal annealing, the peak goes towards larger wave numbers, which indicates an increase in the Si–O–Si bonds; therefore, the oxygen bonds are also increased and it indicates a change in the silicon excess, this makes the stoichiometry of the films closer to that of the SiO_2_ films.

The XPS technique allows information to be obtained on the binding energy of the elements present in a material. From the XPS measurements, the oxygen and silicon composition of the SiO_x_ films and the oxidation states Si^n+^ present were obtained.

**Figure 6:**
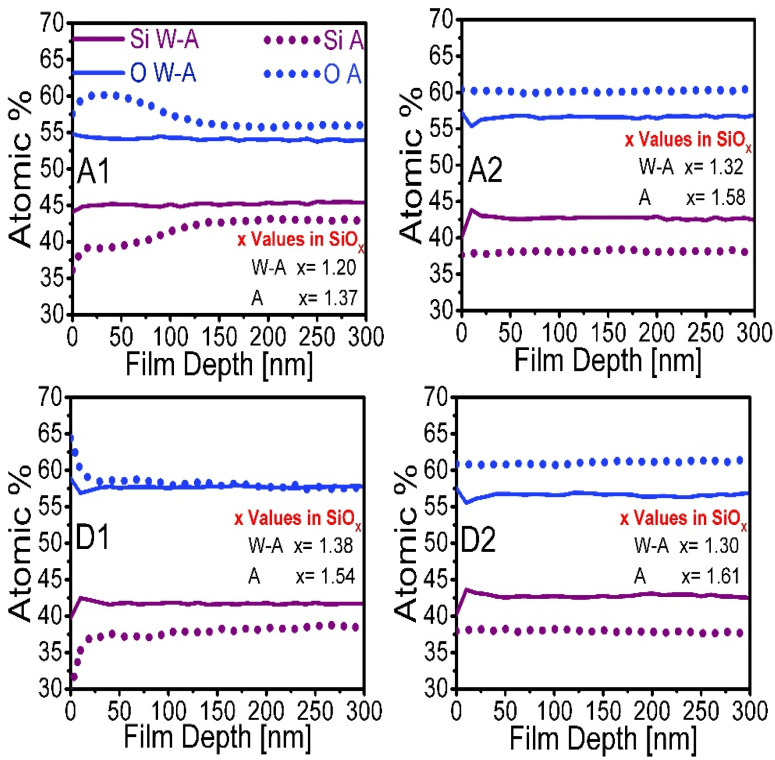
Compositional profile of silicon and oxygen obtained using XPS according to the depth of penetration in the SiO_x_ film and x values in the SiO_x_ stoichiometric ratio.

Figure 6 shows the composition profile in percentage of silicon and oxygen of the films according to the depth of penetration, where dotted line curves are shown for annealed films, while the continuous lines represent films without thermal annealing. With this data it was possible to obtain an average value of x in the stoichiometric SiO_x_ ratio for each of the films using the formula $\frac{O}{Si}=x$ ratio [32].

In Figure 6, a remarkable change is observed in the amount of silicon and oxygen present in the proximity of the film surface with respect to that present inside the volume. Such change is more remarkable for films with thermal annealing. Particularly for the A1 and D2 films, the change is more abrupt. Such films were deposited at a lower ssd; in addition, for these samples the annealing effect enhanced the stoichiometry in the region near the surface due to both the atomic rearrangement and the increment of oxygen atoms. The x values show that in general thermal annealing films present an increase in x, which indicates an increase in the amount of oxygen and a tendency towards SiO_2_ stoichiometry.

To obtain the oxidation states of silicon Si^n+^ (with n = 1, 2, 3, 4) corresponding to the chemical structures Si–OSi_3_, Si–O_2_Si_2_, Si–O_3_Si, and Si-O_4_, respectively [33], as well as the chemical state of silicon in bulk Si0 (Si2p3/2 and Si2p1/2), the XPS spectra of the Si2p peak were deconvolved to find the corresponding binding energies attributed to each of the previous states. These energies are shown in Table 4 [33].

**Figure 7:**
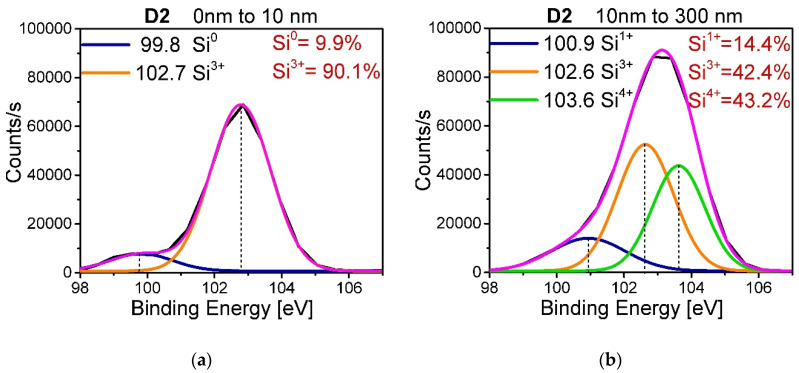
Deconvolution of the D2 film spectra obtained using XPS (**a**) for the region from 0 to 10 nm and (**b**) for the region from 10 to 300 nm.

As an example, in Figure 7 the deconvolution of the spectrum corresponding to the D2 film is shown (a) on the surface of the film (0 to 10 nm) and (b) in the volume of the film (10 nm to 300 nm).

The relative concentration in percentage of each oxidation state (I) can be obtained using Equation (7) [33]:

(7) $$\frac{I^{n+}}{I_{T}}\times 100=I\;\left(n=0,\;1,\;2,\;3,\;4\right)$$

where $I^{n+}$ is the area of the peak that represents the oxidation state Si^n+^, and $I_{T}$ is the total area of the Si2p peak. In Figure 7 it can be seen that for the surface part of the film the binding energies present are attributed to the oxidation states of Si^0^ and Si^3+^, with percentages of 9.9% and 90.1%, respectively, while in volume the Si–Si bonds disappear and the oxidation states Si^1+^, Si^3+^, and Si^4+^ remain, with percentages of 14.4%, 42.4%, and 43.2%, respectively.

The above process was carried out for all the spectra on the surface of the film (10 nm), in the center of the film at a depth of 60 nm, and finally at a depth of 300 nm. The results are shown in Table 5, together with the respective oxidation state attributed to that binding energy.

From Table 5 it is observed that films without thermal annealing present Si^0^ states generally on the surface of the film, except for the A1 film that shows it only in volume, this being also the one with the highest silicon content. Comparing the films according to the ssd of the deposit, films deposited at greater ssd without thermal annealing show the oxidation states Si^0^ and Si^3+^ on the surface of the film, while in volume they have oxidation states Si^1+^, Si^3+^, and Si^4+^. When applying thermal annealing to these films the oxidation states are reduced to Si^3+^ and Si^4+^, both for the surface of the film and for the volume; that is, the stoichiometry of the films is closer to that of the SiO_2_ and the films tend to become uniform in their stoichiometry, in addition the film deposited to greater flow D2’, has a higher percentage of Si^4+^ oxidation state than that deposited at 25 sccm (A2’).

As for the films deposited at lower ssd without thermal annealing, the oxidation state Si^1+^ is absent while the oxidation state Si^2+^ is only present in volume and Si^3+^ is only present on the surface of the sample with lower flux level (25 sccm), but it exists both on the surface and in volume for the sample with a higher flux level (100 sccm), and Si^4+^ exists for both space regions in the two samples. Additionally, the films show significant changes in the percentages of oxidation states only in the most superficial layer. On the other hand, the relevant characteristic of the annealed films is that they present the oxidation states Si^3+^ and Si^4+^ in both space regions; however, the oxidation states Si^0^ and Si^2+^ are absent. The oxidation state Si^1+^ exists on the surface of both samples and in volume but at different depths.

Once the molecular bonds present in the material were identified using the FTIR technique, in addition to the oxidation states being quantified using XPS, it was also necessary to know the structural defects in the SiO_x_ films, for which photoluminescence (PL) measurements were carried out.

**Figure 8:**
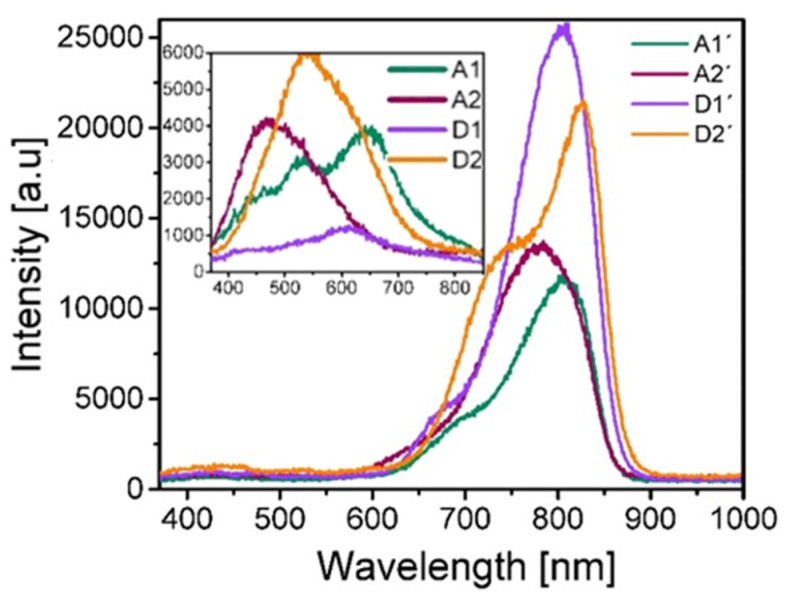
Photoluminescence spectra measured from deposited SiO_x_ films.

The results obtained of such PL measurements are shown in Figure 8, where the inset in the graph depicts the spectra obtained from the films without thermal annealing, while the main graph shows the PL spectra obtained from the annealed films. It is worth noting that the PL intensity from all films with thermal annealing is augmented approximately up to 4 times with respect to that of the films without thermal annealing. Additionally, such spectra undergo a shift in the emission wavelength towards lower energies in such a way that PL intensity lies in the range 650–900 nm. It should be noted that the strongest PL intensity corresponds to the annealed films deposited at a flow level of 100 sccm, while for films without thermal annealing it corresponds to the films deposited at flow levels of 100 sccm and 25 sccm, and the emission region is wider, in the range 400–800 nm. It is noticeable that all the emission PL bands are wide, which indicates that they are composed of several emission peaks that have a shape similar to a Gaussian one.

**Figure 9:**
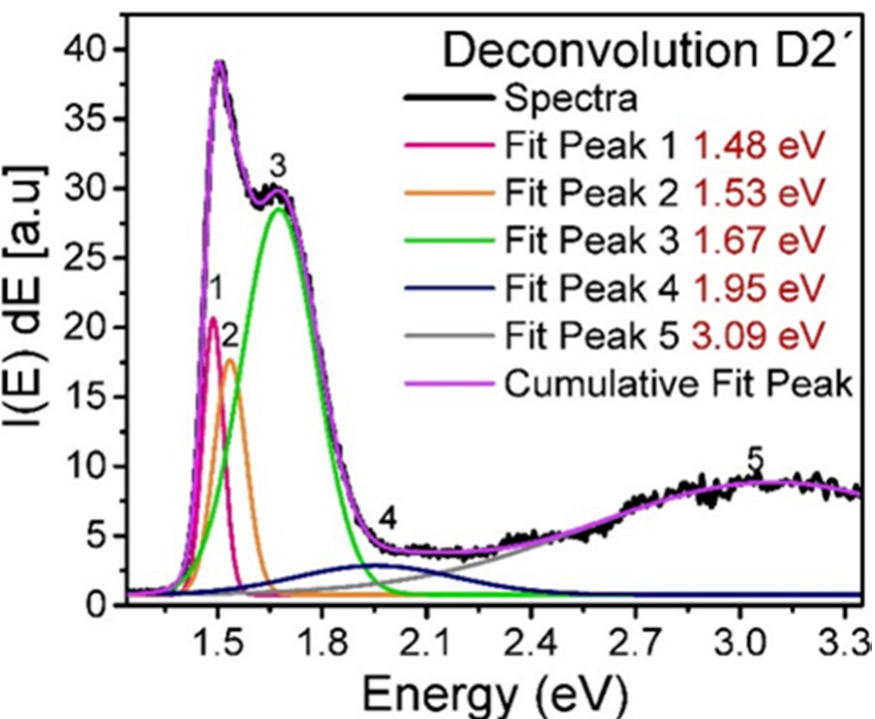
Photoluminescence spectrum deconvolved for the D2’ film.

For SiO_x_, the most accepted light emission mechanisms which give rise to the visible and near infrared bands are attributed to the following causes: the effect of dimensional quantum confinement in Si-ncs or silicon nanoclusters (Si-ncl) whose reported photoluminescence lies in the emission energy range from 1.3 eV to 1.7 eV [34,35,36] and the effect related to defects in the oxide matrix such as weak oxygen bonds (WOB) with a PL peak at 3 eV [36], neutral oxygen vacancy (NOV) with PL ranging from 2.8 eV to 2.9 eV [37], hydrogen-related defects (H) with PL located in the range from 2.2 eV to 2.5 eV [38], and finally a non-bridging oxygen hole center (NBOHC) with PL from 1.8 eV to 2 eV [39,40]. These emission mechanisms make the PL spectra exhibit a wide shape, as can be seen in Figure 8. In order to find all possible contributions to the photoluminescent processes in the SiO_x_ films, the deconvolution of each spectrum was performed, by way of example. Figure 9 depicts the deconvolution of the photoluminescent spectrum of the D2’ film. To perform the deconvolution and in order to avoid common errors reported in the photoluminescence analysis, the spectra were deconvolved using the PL intensity-energy differential versus energy [41].

Table 6 shows the position of the peaks obtained from the deconvolution of the photoluminescent spectra, in addition to the defects to which the emission is attributed, according to their energy position.

As can be seen in Table 6, films without thermal annealing show photoluminescence attributed to NOV and H, in addition to films deposited at a shorter ssd showing the presence of NBOHC, while those deposited at a larger ssd have WOB. For annealed films, photoluminescence is mainly due to Si-ncl effects and to a lesser extent to NBOHC and WOB defects.

## 4. Discussion

**Figure 10:**
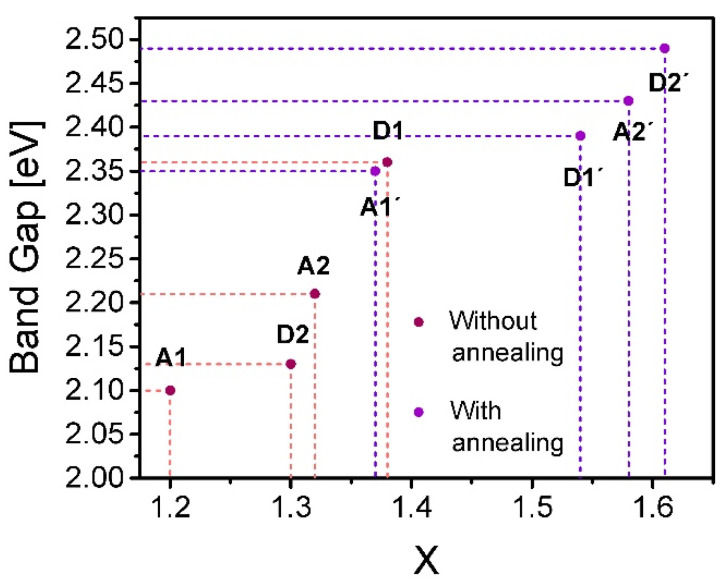
Correlation between the stoichiometric ratio x and the calculated energy band gap values.

**Figure 11:**
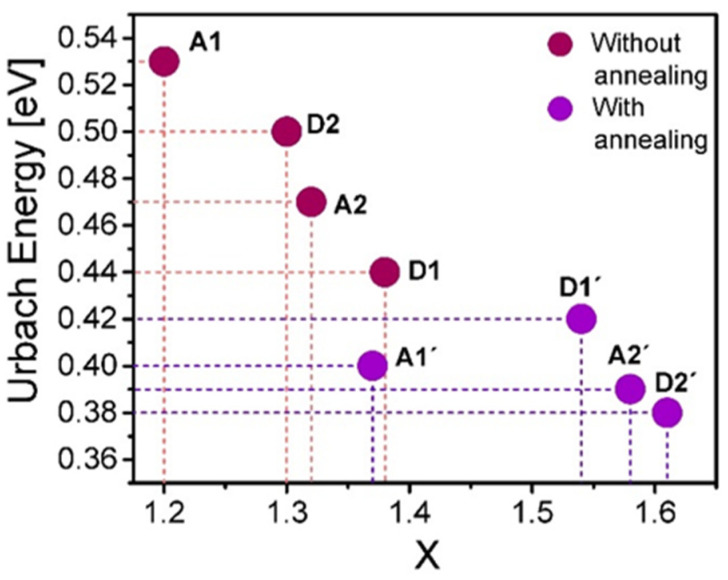
Correlation between the stoichiometric ratio x and the calculated Urbach energy values.

Using the calculated values of the BG, the UE parameters, and making a correlation with the results obtained using XPS, we identify a clear dependence between the stoichiometric ratio (x) and the BG and UE values. Figure 10 and Figure 11 display how the correlation between the stoichiometric ratio and BG and UE evolves.

Figure 10 shows a clear tendency in films in which with a higher silicon or lower oxygen content (lower value of x) the lower BG is obtained. Such a trend is held both with and without annealing, and it is relevant to point out that the BG increases significantly due to both thermal annealing and the increase of oxygen content. In addition, the UE decreases monotonously in accordance with the silicon content decreasing with the thermal annealing, as is shown in Figure 11. Special attention deserves to be paid to the fact that the annealed films with the larger ssd and higher flow level offer the highest BG and stoichiometric ratio and the lowest UE.

The calculated Urbach energies occupy a wide range of values ranging from 380 meV to 530 meV. These are considerable energy values, and it indicates in some way the level of structural disorder that exists in the SiO_x_ films. This disorder is remarkably diminished both with the oxygen increment and with the thermal annealing. In addition to that the dependence among the stoichiometric ratio, band gap and Urbach energies is well defined and agrees with the model established by Mott [31]. For values of the stoichiometry ratio x further from that of the stable configuration of silicon dioxide, the films undergo a greater disorder in the molecular structure of the material, which brings the increment of aggregate energy levels inside the intrinsic band gap energy of the dioxide matrix, causing the formation of wider band tails and consequently a smaller band gap energy that tends towards that of the silicon one.

**Figure 12:**
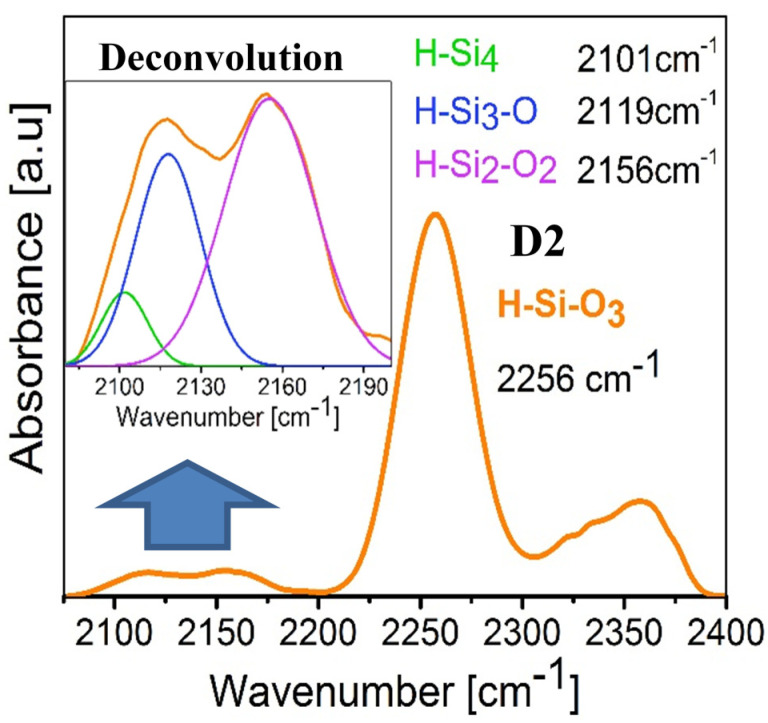
FTIR spectrum of the D2 film showing the absorption peaks attributed to the different configurations of the Si-H bonds.

Comparing these results with the deposition parameters, we find a behavior in which, with a lower flow level and shorter ssd, films with a higher silicon content (lower BG and higher UE) are obtained, and therefore, the optical response of the material is substantially modified. It is worthwhile to note that the trend in which the flow level and ssd determine the dependence between the silicon content and BG and UE is not well defined for the case of the films without thermal annealing, contrary to the case of the annealed films for which the increment of the ssd and flow level diminish the silicon content and increase the BG and reduce the UE clearly. Furthermore, for the films without thermal annealing, the presence of hydrogen is observed in the FTIR spectra of these films (see Figure 12), which suggests that hydrogen plays an important role which causes a clear dependence between the silicon content and the GB and UE parameters not to exist, as in the case of the annealed films in which hydrogen is no longer present.

Figure 12 shows an amplification of the IR spectrum in the region from 2100 cm^−1^ to 2400 cm^−1^ for the D2 film; in addition, the deconvolution made from the 2075 cm^−1^ peak to the 2150 cm^−1^ one is shown. Such deconvolutions were performed to observe the absorption peaks attributed to the Si–H bonds in the H–Si≡Si_3_, H–Si≡O_2_ and H–Si≡O_1_ configurations, while Table 7 lists the peak positions for all films without thermal annealing, which are the ones that present the Si–H bonds.

Figure 12 shows the presence of the H–Si≡Si_3_, H–Si≡O_2_, and H–Si≡O_1_ configurations, whose contributions are minimal compared to the one due to the H–Si≡O_3_ configuration, which is found at position 2256 cm^−1^ and shows a significant right shoulder which is ascribed to an effect of the first neighbors with silicon atoms [30]. When comparing these results with those obtained using XPS, where it is observed that the predominant oxidation states are Si^3+^ and Si^4+^, t is then confirmed that an important part of these Si≡O_3_ bonds are bound to hydrogen atoms.

Regarding the hydrogen content in the films without thermal annealing, it is greater for films deposited at a larger ssd. This is explained by indicating that a greater deposit distance leads to the formation of weak silicon-silicon or silicon-oxygen bonds. These bonds, as it is well known, are broken by the action of hydrogen forming covalent bonds Si–H, which explains the presence of the H–Si≡O_3_, H–Si≡Si_3_, H–Si≡O_2_, and H–Si≡O_1_ configurations in the FTIR spectra.

The results obtained using PL are used in the analysis of the formation of defects in the SiO_x_ films; however, it is hitherto the most controversial and difficult topic without conclusive reasons. In the case of the results obtained for films deposited without thermal annealing, the abovementioned result is shown regarding the presence of weak oxygen bonds, which are shown for films deposited at a greater ssd. Neutral oxygen vacancies are present for all films without thermal annealing, which is common for this type of material.

The emission related to defects involving hydrogen is more evident for films deposited at a larger ssd (A2, D2). In the case of NOVs, it is known that they react with hydrogen to form H–Si≡ configurations, so it is interesting to note that this defect is only present in films without thermal annealing. It is very probable that films without annealing have converted a part of their NOVs into H–Si≡O_3_ configurations.

Since the films without thermal annealing deposited at a shorter ssd have a higher silicon content and oxidation states Si^0^ with respect to the other films, we expect that the emission at 1.9 eV is not mostly due to NBOHC defects that are closely related to the SiO_4_, but to another type of defect which is also reported with this characteristic emission, interstitial oxygen ($O_{2}^{-},O_{3}^{-}$), which would be more appropriate since NBOHC is not present in the A2 and D2 films that have a greater amount of Si^4+^ oxidation states; however, it is not discarded that it is a contribution of both emission mechanisms.

In the case of PL in annealing films, they all show the same emission mechanisms, attributed to WOB, NBOHC, and the greater contribution to PL is attributed to Si-ncl effects, which brings confinement effects due to a nanocrystal formation producing an increase of the band gap, which is controlled by the Si nanostructure size [42]; however, it is believed that there is a better explanation for this purpose, which will be discussed later, in addition to the fact that emission intensity increases substantially compared to that of films without thermal annealing, which indicates that the structural rearrangement of the material is closely related to this reported emission. To understand how the material restructuring occurs, we take into account the results obtained from the deconvolution of the Si2p peak, as mentioned by Tomozeiu [31]. Based on the Gibbs free energy calculations, it has been shown that the bonds Si-(Si_4_) and Si-(O_4_) are stable, while the Si-(Si_n_O_4−n_) bonds, with n = 1, 2, 3 are unstable, being Si-(Si_2_O_2_), which corresponds to a state of Si^2+^ oxidation, the less stable structural entity. The films deposited at an ssd of 5 mm without thermal annealing present this oxidation state, which leads to a greater tension or stress of the chemical bond between the central silicon atom and the oxygen ones, so if the conditions for the migration of an oxygen atom are satisfied, a phase decomposition will occur following the next reaction:

Si-(Si_2_O_2_) + Si-(Si_2_O_2_) → Si-(Si_1_O_3_) + Si-(Si_3_O_1_)

Si-(Si_2_O_2_) + Si-(Si_2_O_2_) → Si-(O_4_) + Si-(Si_4_)

The first reaction is observed according to the oxidation states present when applying thermal annealing to these films (Si^1+^ and Si^3+^). However, in the films deposited at a larger ssd, the states Si^1+^ and Si^3+^ are already present in the films without thermal annealing, indicating that the deposition of the film under this condition leads to a more stable lattice growth that could be favored as the results of FTIR indicate. Due to the incorporation of hydrogen and the formation of a greater amount of H–Si≡O_3_ bonds, where hydrogen helps to reduce tension in the molecular structure, the presence of hydrogen could be inferred not only using this technique, but could also be considered through the photoluminescent emission band (2.2 eV to 2.5 eV) attributed to defects related to hydrogen [40]. When applying thermal annealing to these films as observed using FTIR, a hydrogen desorption occurs so that the H–Si≡O_3_ bond can become a Si-(SiO_3_) bond with the addition of a silicon atom. It can be observed in Table 6 that when the films were annealed, only the oxidation states Si^3+^ and Si^4+^ survived, and such states are present in the A2’ and D2’ films that have a stoichiometry closer to that of SiO_2_ and a major BG and a smaller UE.

**Figure 13:**
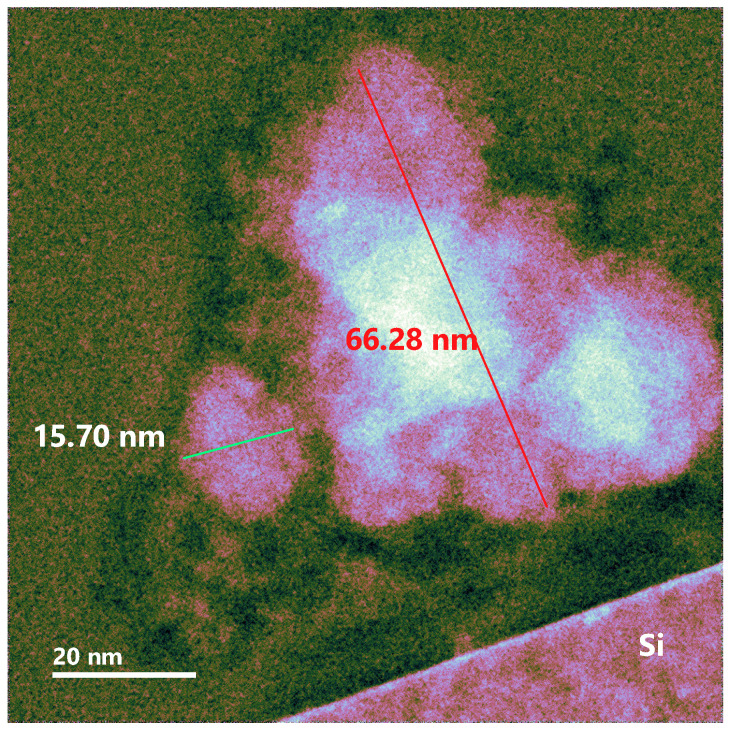
High Resolution Transmition Electronic Microscopy micrograph of the A1 SiO_x_ film.

**Figure 14:**
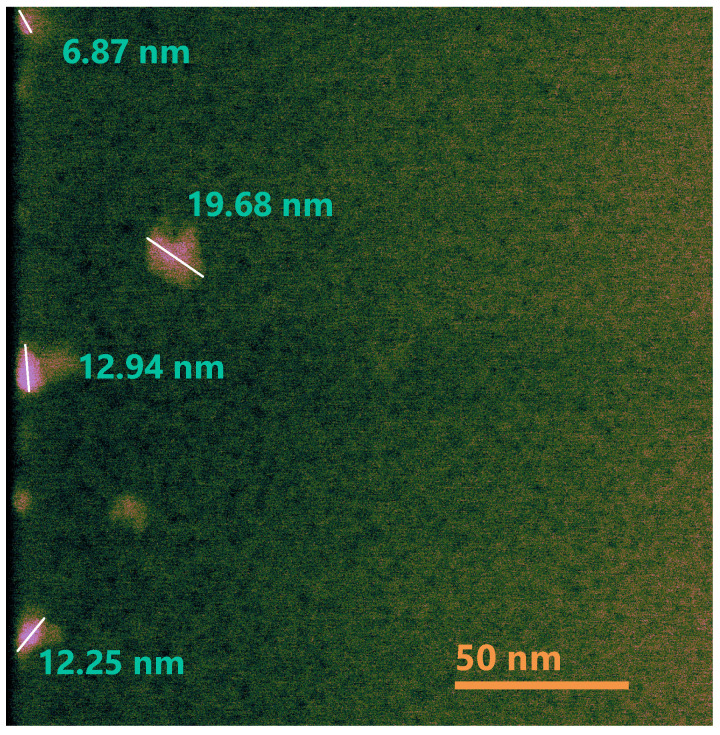
HRTEM micrograph of the A2 SiO_x_ film.

As previously mentioned, reports that exist regarding the structural arrangements that are presented when thermal annealing is performing in this type of films suggest, given the stoichiometry, the formation of isolated nanoclusters or nanocrystals of silicon immersed in a SiO_2_ matrix, instead of an arrangement of the type of percolated networks or Si-SiO_2_ sponges that are formed when there is an excess of Si greater than 30% [20,43]. One method to characterize these silicon nanocrystals or nanoclusters is through the XRD technique; however, a difficulty has been reported in obtaining a diffraction pattern for very small Si nanocrystals dispersed in the amorphous SiO_x_ matrix due to the fact that they have a short-range crystalline order [44]; therefore, to complement this study, the HRTEM technique was carried out in order to corroborate the presence of Si-ncs or Si-ncls. The HRTEM micrographs for films A1 and A2 are shown in Figure 13 and Figure 14, respectively. These figures show the presence of silicon clusters. In Figure 13, film A1 has silicon clusters of different sizes and has the largest clusters, which corresponds to what was observed using XPS regarding the presence of Si^0^ states; in addition, this film has a greater excess of silicon. In the case of Figure 14, which shows the micrograph of film A2, the presence of silicon clusters can be seen in the same way, but in this case with a smaller size.

**Figure 15:**
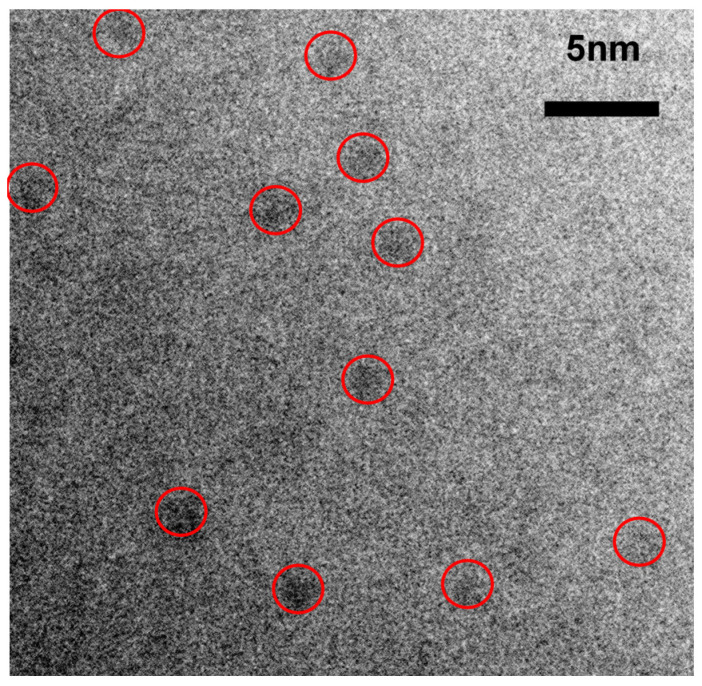
HRTEM micrograph of the D1SiO_x_ film.

**Figure 16:**
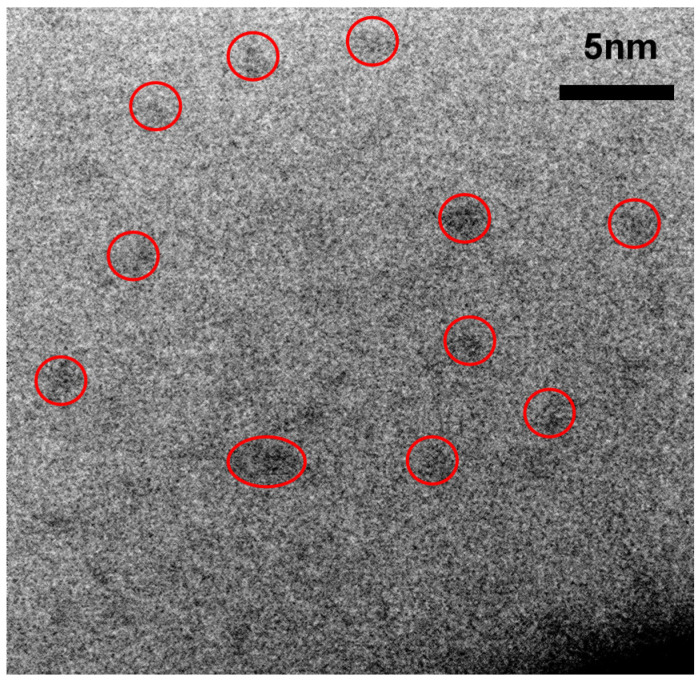
HRTEM micrograph of the D2 SiO_x_ film.

The HRTEM micrographs for films D1 and D2 are shown in Figure 15 and Figure 16, respectively. These figures show the presence of dark spots that could represent silicon clusters in which the crystalline orientation was not observed (Si-ncls). They have an average size of 3 nm, which corroborates what was inferred using XPS with respect to the existence of a greater silicon concentration (Si^0^ state) in films without thermal annealing.

**Figure 17:**
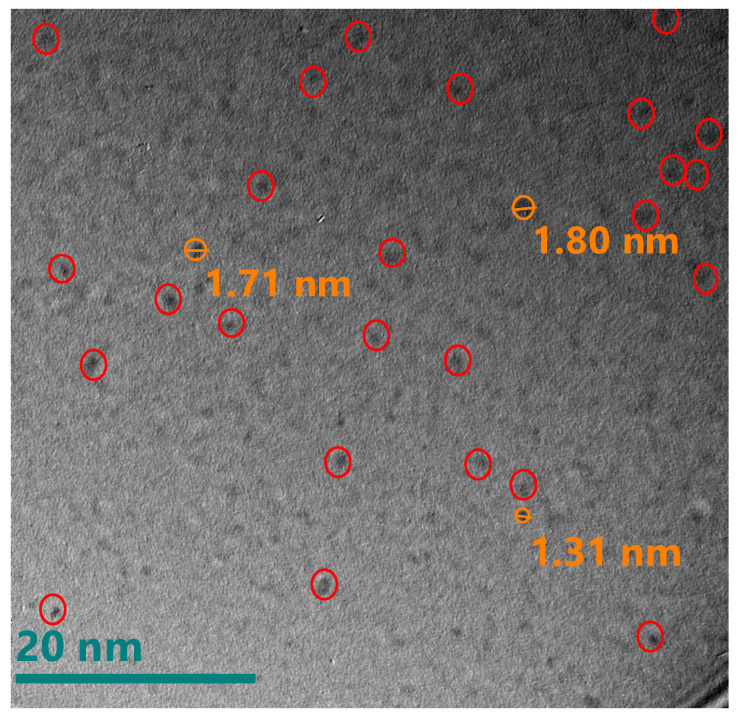
HRTEM micrograph of the D1’ SiO_x_ film.

**Figure 18:**
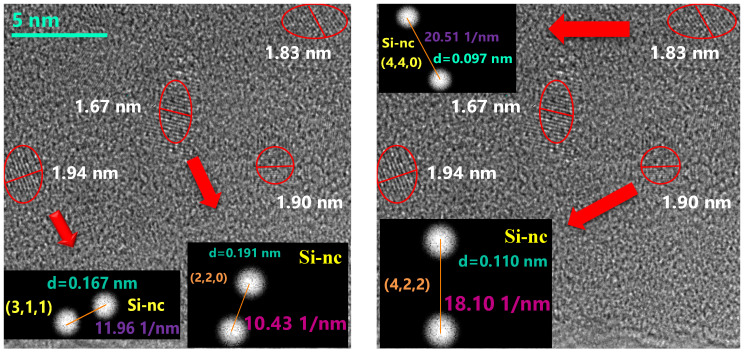
HRTEM micrograph of the D1’ SiO_X_ film with crystallographic orientations.

Figure 17 and Figure 18 show the HRTEM micrographs of the D1’ fil., Figure 17 shows a large number of clusters with a diameter <2 nm, while Figure 18 shows an enlarged micrograph showing certain regions with dimensions between 1 nm and 2 nm that show a certain crystalline orientation. To obtain the crystallographic orientation, the interplanar distances were measured. The micrographs shown in Figure 18 were analyzed using a Digital Micrograph program using Fourier transform to obtain the reciprocal space and thus be able to obtain the interplanar distances. With the interplanar distances, we proceeded to search the orientation of the nanocrystals by means of the crystallographic data provided by the database PDF 4. The presence of crystalline silicon with reference code 00-027-1402 is appreciated.

Correlating the results obtained from the various characterizations performed and the micrographs shown, it is important to highlight that the presence of the Si^0^ state in all the films without thermal annealing is due to the formation of Si-ncls with sizes greater than 10 nm. In the case of the film A1 which is the one with the highest excess of silicon corresponding to the one that reached the highest temperature of the deposit and has the lowest flow, it also presents Si-ncls of greater size as shown in Figure 13. The existence of the state Si^0^ in the SiO_x_ films, as can be seen in the phase decomposition, generally occurs after samples are subjected to a thermal annealing above 1000 °C [31]. This thermal process provokes the formation of Si-ncs in a SiO_2_ matrix. With this process the material reaches a stable configuration; however, in this case, when applying thermal annealing, the state Si^0^ disappears. This may be due to the resolution reached from the XPS measurement in depth, which may be less than what is required to detect Si-ncs of sizes less than 10 nm.

As previously mentioned, the band of PL emission in the range from 1.3 eV to 1.7 eV is attributed by several authors to the effect of quantum confinement in Si-ncs or silicon Si-ncls [34,35,36]; however, in this theory, the size of the nanocrystal determines the color of the emission. In larger nanocrystals, the emission tends to lower energies, and as we can see from the results, the Si-ncs and Si-ncls are of a size that would correspond to a blue emission. These results make us take into account what is reported by other authors [35,45] who suggest that the intense emission around 1.5 eV is caused by small, oxidized silicon grains (1 nm or 2 nm). According to a model of molecular type emitters at the Si/SiO_2_ interface in the work of Guerra and Ossicini [45], the importance of oxidation in nanocrystals for emission is shown, and it is corroborated that those mostly oxidized have a high photoluminescence. This could be compared with that obtained for porous silicon oxidized by Wolking et al. [46]. In their report it is corroborated that for clusters greater than 3 nm the behavior attributed to quantum confinement (QC) is fulfilled and that when the cluster size is smaller the emission intensity results are greater. In this, case the emission process is attributed to the recombination of the free excitons. For clusters between 2 and 3 nm, the previous behavior is still satisfied but not to the same rate in which it is fulfilled in QC. Here, the recombination is related to a free hole and a trapped electron, and finally, for clusters less than 2 nm, an emission redshift occurs and the recombination processes are attributed to trapped excitons.

Given the size observed in Figure 17 and Figure 18, it could be suggested that the thermal annealing in the films induces a phase separation that further reduces the size of the Si-ncl. In addition, by virtue of the presence of interstitial oxygen in the films without annealing, it is possible that an oxidation process occurs of these small, silicon nanocrystals in the Si-nc/SiO_2_ interface. Such an event may explain why the PL emission shifts towards red. In addition to the other experimental results obtained regarding the increase in the amount of oxygen in the films and the rearrangement of the molecular network, this would corroborate the theory presented in the literature [35,45].

## 5. Conclusions

Using the spectroscopic ellipsometry technique, the transmittance, reflectance, and absorption coefficient of the films were calculated, with which it was subsequently possible to calculate the values of BG and UE. Such parameters show a tendency by which, at a higher oxygen content, the value of BG increases, while the value of the UE decreases. Similarly, the films with thermal annealing present a decrease in the UE, which suggests a decrease in the molecular structure disorder and the level of defects. By means of the XPS technique, it was possible to obtain an approximate value of the stoichiometric ratio for deposited SiO_x_ films whose values varied between 1.2 and 1.61, as well as the oxidation states of silicon. It was observed that films deposited at a higher ssd show a greater value of x in the stoichiometric ratio, possibly due to a greater incorporation of hydrogen, which was sustained according to what was observed using FTIR. From the results of PL, the bands attributed to the main defects observed in the SiO_x_ were found. Thus, in the case of films without thermal annealing, the violet band is ascribed to WOB defects, the blue band to NOV defects, the green band to H, and the orange band to NBOHC, while in the case of the annealed films, they exhibited the emission attributed to WOB, NBOHC, and quantum confinement effects (with emission in red), due to silicon nanocrystals. This contradicts the results obtained using XPS where the concentration of silicon decreases with thermal annealing, which leads us to suggest the presence of small, oxidized silicon grains (less than 2 nm) according to the results obtained, which were corroborated using HRTEM. In addition, Si-ncls were obtained without applying thermal annealing to the deposited films, while the annealing induces a phase separation that dramatically increases the PL, which could be an advantage for the numerous potential applications where silicon agglomerates with small diameters enrich the luminescent properties of the material.

## Figures and Tables

**Table 1:** Description of the labeling of the eight SiO_x_ films obtained.

	Hydrogen Flow Level: 25 sccm		Hydrogen Flow Level: 100 sccm	
Source-Substrate Distance:	Without Annealing	With Annealing	Without Annealing	With Annealing
**5 mm**	A1	A1’	D1	D1’
**8 mm**	A2	A2’	D2	D2’

**Table 2:** Energy band gaps and Urbach energies.

**Hydrogen flow: 25 sccm**		
	**A1**	**A2**
**Without annealing**	**2.1 eV**	**2.21 eV**
	UE: 0.53 eV	UE: 0.47 eV
**With annealing**	**2.35 eV**	**2.43 eV**
	UE: 0.40 eV	UE: 0.39 eV
**Hydrogen flow: 100 sccm**		
	**D1**	**D2**
**Without annealing**	**2.36 eV**	**2.13 eV**
	UE: 0.44 eV	UE: 0.50 eV
**With annealing**	**2.39 eV**	**2.49 eV**
	UE: 0.42 eV	UE: 0.38 eV

**Table 3:** Peak positions present in the spectra of Figure 5, as well as the vibrational modes and the type of molecule to which they are attributed.

	A1	A2	D1	D2	A1’	A2’	D1’	D2’
**Rocking Si–O–Si**	451 cm^−1^	453 cm^−1^	451 cm^−1^	455 cm^−1^	461 cm^−1^	461 cm^−1^	463 cm^−1^	463 cm^−1^
**Bending Si–O–Si**	808 cm^−1^	802 cm^−1^	802 cm^−1^	800 cm^−1^	812 cm^−1^	812 cm^−1^	812 cm^−1^	812 cm^−1^
**Stretching Si–O–Si**	1074 cm^−1^	1068 cm^−1^	1070 cm^−1^	1060 cm^−1^	1089 cm^−1^	1089 cm^−1^	1091 cm^−1^	1091 cm^−1^
**Bending H–Si≡O_3_**	883 cm^−1^	881 cm^−1^	881 cm^−1^	879 cm^−1^	-		-	-
**Stretching H–Si≡O_3_**	2258 cm^−1^	2258 cm^−1^	2256 cm^−1^	2256 cm^−1^	-		-	-

**Table 4:** Binding energies of the Si core levels and the Si oxidation states.

	Si2p_3/2_	Si2p_1/2_	Si^1+^	Si^2+^	Si^3+^	Si^4+^
**Binding energies**	99.5 eV	100 eV	101 eV	101.5 eV	102.5 eV	103.5 eV

**Table 5:** Binding energies of the decomposed XPS peaks.

	Si^0^	Si^1+^	Si^2+^	Si^3+^	Si^4+^		Si^0^	Si^1+^	Si^2+^	Si^3+^	Si^4+^
**A1** 10 nm	-	-	-	102.5 eV 10.6%	104.5 eV 89.4%	**A2** 10 nm	99.7 eV 12.3%	-	-	102.8 eV 87.7%	-
**A1** 60 nm	99.9 eV 9.3%	-	101.8 eV 33.4%	-	103.2 eV 57.3%	**A2** 60 nm	-	101.2 eV 12.4%	-	102.5 eV 40%	103.5 eV 47.6%
**A1** 300 nm	99.9 eV 9.3%	-	101.8 eV 33.4%	-	103.2 eV 57.3%	**A2** 300 nm	-	101.2 eV 12.4%	-	102.5 eV 40%	103.5 eV 47.6%
**A1’** 10 nm	-	101.2 eV 12.5%		102.4 eV 38%	103.3 eV 49.5%	**A2’** 10 nm	-	-	-	102.6 eV 64%	103.7 eV 36%
**A1’** 60 nm	-	-		102.6 eV 25.8%	103.7 eV 74.2%	**A2’** 60 nm	-	-	-	102.6 eV 64%	103.7 eV 36%
**A1’** 300 nm	-	101 eV 16.5%		102.6 eV 48.5%	103.7 eV 35%	**A2’** 300 nm	-	-	-	102.6 eV 64%	103.7 eV 36%
**D1** 10 nm	99.5 eV 7.4%	-	-	102.3 eV 38.6%	103.2 eV 54%	**D2** 10 nm	99.8 eV 9.9%	-	-	102.7 eV 90.1%	-
**D1** 60 nm	-	-	101.5 eV 18.1%	102.5 eV 40.1%	103.8 eV 40.8%	**D2** 60 nm	-	100.9 eV 14.4%	-	102.6 eV 42.4%	103.6 eV 43.2%
**D1** 300 nm	-	-	101.5 eV 18.1%	102.5 eV 40.1%	103.8 eV 40.8%	**D2** 300 nm	-	100.9 eV 14.4%	-	102.6 eV 42.4%	103.6 eV 43.2%
**D1’** 10 nm	-	101.2 eV 14.8%	-	102.8 eV 45.2%	103.8 eV 40%	**D2’** 10 nm	-	-	-	102.5 eV 39.3%	103.5 eV 60.7%
**D1’** 60 nm	-	101.2 eV 17.4%	-	102.8 eV 43.8%	103.7 eV 38.8%	**D2’** 60 nm	-	-	-	102.5 eV 38%	103.6 eV 62%
**D1’** 300 nm	-	-	-	102.5 eV 43.7%	103.8 eV 56.3%	**D2’** 300 nm	-		-	102.5 eV 38%	103.6 eV 62%

**Table 6:** PL deconvoluted bands and their proposed defects contributions.

	A1	A1’	A2	A2’		D1	D1’	D2	D2’
**WOB**	-	3.09 eV	3.17 eV	3.3 eV	**WOB**	-	3.15 eV	3.1 eV	3.09 eV
**NOV**	2.79 eV	-	2.82 eV	-	**NOV**	2.90 eV	-	2.82 eV	-
**H**	2.34 eV	-	2.53 eV	-	**H**	2.40 eV	-	2.32 eV	-
**NBOHC**	1.95 eV	2.04 eV	-	1.82 eV	**NBOHC**	2.03 eV	2.04 eV	-	1.95 eV
**Si-ncl**	-	1.51 eV 1.58 eV 1.74 eV	-	1.53 eV 1.63 eV	**Si-ncl**	-	1.51 eV 1.59 eV 1.76 eV	-	1.48 eV 1.53 eV 1.67 eV

**Table 7:** Si–H stretching vibration bands.

	A1	A2	D1	D2
**H–Si≡Si**	2100 cm^−1^	2097 cm^−1^	2100 cm^−1^	2101 cm^−1^
**H–Si≡O_1_**	2117 cm^−1^	2108 cm^−1^	2115 cm^−1^	2119 cm^−1^
**H–Si≡O_2_**	2154 cm^−1^	2150 cm^−1^	2165 cm^−1^	2156 cm^−1^
**H–Si≡O_3_**	2258 cm^−1^	2258 cm^−1^	2256 cm^−1^	2256 cm^−1^
